# Depression and Associated Factors among Adult Inpatients at Public Hospitals of Harari Regional State, Eastern Ethiopia

**DOI:** 10.1155/2018/6743520

**Published:** 2018-04-01

**Authors:** Haile Tilahun, Nefsu Awoke, Biftu Geda, Firehiwot Mesfin

**Affiliations:** ^1^Hiwot Fana Specialized Teaching Hospital, College of Health and Medical Sciences, Haramaya University, Harar, Ethiopia; ^2^Department of Nursing, College of Health Science and Medicine, Wolaita Sodo University, Wolaita Sodo, Ethiopia; ^3^School of Nursing and Midwifery, College of Health and Medical Sciences, Haramaya University, Harar, Ethiopia; ^4^School of Public Health, College of Health and Medical Sciences, Haramaya University, Harar, Ethiopia

## Abstract

**Introduction:**

Globally, depression is one of the three leading causes of disease and it will be the second leading cause of world disability by 2030. The prevalence of depression in Sub-Saharan Africa ranges from 15 to 30%. In Ethiopia, depression was found to be the seventh leading cause of disease burden and its prevalence has been increased in hospital compared to community setting because hospital environment itself is stressful. Yet, no study was done in Eastern Ethiopia, where substance use like Khat is very rampant.

**Objective:**

To assess depression and associated factors among adult inpatients at public hospitals of Harari Regional State, Eastern Ethiopia, from February 01 to 28, 2017.

**Methodology:**

Hospital based cross-sectional study design was employed on 492 admitted adult patients in Harari region hospitals. Consecutive sampling method was used to include study population. The data were collected by interviewee and analyzed by SPSS version 20.0. Bivariate and multivariate logistic regression analyses were employed. *p* value of 0.05 or less was considered to be statistically significant.

**Result:**

A total of 489 patients were interviewed with response rate of 99.4%. Having duration of 1-2 weeks in the hospital [AOR = 2.02, 95% CI: (1.28, 3.19)], being diagnosed with chronic morbidity [AOR = 4.06, 95% CI: (2.23, 7.40)], being users of psychoactive drugs [AOR = 2.24, 95% CI: (1.18, 4.24)], and having been admitted to surgical ward [AOR = 0.50, 95% CI: (0.31, 0.81)] were significantly associated with depression.

**Conclusion and Recommendation:**

Prevalence of depression among admitted inpatients was high. Therefore, increasing the awareness of benefits of early diagnosis of patients to prevent major form of depression and strengthening the clinical set-up and establishing good referral linkage with mental health institutions was considered to be cost-effective method to reduce its prevalence.

## 1. Introduction

Depression or depressive disorders are mental illnesses characterized by a profound and persistent feeling of sadness or despair and/or a loss of interest in things [1]. Everyone experiences feelings of unhappiness and sadness occasionally, but when these depressed feelings start to dominate everyday life and cause physical and mental deterioration, they become what are known as depressive disorders [2].

World Health Organization (WHO) reported that 350 million people are affected by depression with prevalence of 3–16.9% throughout the worldwide [3]. The World Mental Health Survey reported that 15% of the population from high-income countries compared to 11% from low- and middle-income countries was likely to get depression over their life time [4]. Approximately 60 million people in the United States live with one of four chronic conditions of which major depression is common [5].

Globally, depression is one of the three leading causes of disease and it will be the second leading cause of world disability by 2030 [6]. It is the largest contributor of disease burden [4]. Therefore, failure to recognize and treat depression endangers the patient as well as the community at large [7].

Depression is a common and costly comorbidity among people with diabetes and another chronic disease [8] and it increases use of healthcare services and expenses and can result in early death and disturbance in the general state of wellness [1]. An analysis of USA national claims data for more than 9 million people showed that patients with chronic physical disease who were also receiving treatment for depression or anxiety had average monthly medical costs that were between 33% and 169% higher over a range of conditions and these costs excluded expenditure on mental health services [9].

Depression increases the risk of CHF particularly in those already at risk for CHF (e.g., patients with systolic hypertension) [10]. Depression is also a strong risk factor for normal subjects progressing to mild cognitive impairment [11]. It also causes disability of functional impairment, decreased quality of life, has a negative effect on the body's recovery from illness, and increases the rate of suicide [1].

The prevalence of depression in Sub-Saharan Africa ranges from 15 to 30% [12]. In Nigeria, it ranges between 17% and 27% among hospitalized patients [13]. In Ethiopia, depression was found to be the seventh leading cause of disease burden [14] and prevalence of depression has been increased in hospital compared to community setting because hospital environment itself can be stressful [4].

However, in Eastern Ethiopia, where substance use like Khat is very rampant, study related to magnitude of depression and associated factors among adult inpatients is not done yet. Therefore the aim of this study is to minimize this information gap in order to be used as a baseline for other researchers.

### 1.1. Significance of the Study

The aim of this study is to investigate the prevalence of depression among admitted adult patient and to extend understanding on the relationship of factors that lead to depression. The primary beneficiaries of the study were patients by helping them to understand the factors that lead to depression and take preventive actions to reduce its rate. Additionally this study helps healthcare providers to initiate the early diagnosis and management of depression based on research finding. The finding of this research may help the hospitals to know the prevalence of depression and understand factors associated with it and to intervene accordingly to reduce its prevalence.

The study may help policy makers at national level and other none governmental organization (NGOs) by providing relevant information for the future planning and intervention. Also it will be used as input for further research to compare the rates of performance. As nurses have more contact with patients than any other healthcare provider, the important role of nursing in addressing the mental health needs of patients across all healthcare settings is clearly crucial to its holistic philosophy. Indeed, if the nursing profession is to uphold this philosophy as more than just rhetoric, the meeting of mental healthcare needs must be embraced enthusiastically.

### 1.2. Objectives

#### 1.2.1. General Objective

The main objective of this study is to assess depression and associated factors among adult inpatients at public hospitals of Harari Regional State, Eastern Ethiopia, from February 01 to 28, 2017.

#### 1.2.2. Specific Objectives

- To determine the prevalence of depression among adult inpatients at public hospitals of Harari Regional State
- To identify factors associated with depression among adult inpatients at public hospitals of Harari Regional State

## 2. Methodology

Hospital based cross-sectional study design with quantitative method was conducted in two public hospitals of Harari Regional State (i.e., Hiwot Fana Specialized University Hospital (HFSUH) and Jugal Hospital) from February 01 to 28, 2017. All adult patients who were aged 18 years and above and were admitted to the medical, surgical, and gynecological wards were interviewed by the trained data collectors using structured and pretested questionnaires to assess the presence of depression and patients who are already under treatment of depression were excluded.

The data collection tool was developed by reviewing different literature which contains four parts: Part I: sociodemographic factors, Part II: type of diagnosis, Part III: substance abuse, and Part IV: Patient Health Questionnaire-9 (PHQ-9) which was used to rate level of depression.

Depression was assessed using the nine-item Patient Health Questionnaire-9 (PHQ-9), which was prepared in English and translated to local languages (Amharic and Afan Oromo).

Data were entered and edited into EPI-data version 3.1 and cleaned and analyzed by using SPSS for windows program version 20. Descriptive statistics of different variables were determined and the results were presented in texts, tables, and graphs using summary measures such as percentages, mean, and median. Hosmer-Lemeshow's and Omnibus tests were done to test for model fitness. Bivariate logistic regression was carried out to identify the associated factors with depression. All variables with *p* value ≤ 0.25 were taken into consideration in the multivariable model to control for all possible confounders and the variables were selected by enter method to see the effect of each variable on the outcome variable. Multicollinearity tests were carried out to see the correlation between independent variables using standard error and one of the independent variable was dropped for those with standard error of >2. Finally the results of multivariable logistic regression analysis were presented in crude and adjusted odds ratio with 95% confidence intervals. Level of statistical significance was declared at *p* value < 0.05.

The study protocols, informed consent forms, and data collection instruments were approved by College of Health and Medical Sciences Institutional Health Research Ethics Review Committee. Official letters of cooperation were written to HFSUH and Jugel Hospital and concerned bodies to obtain their cooperation in facilitating the study. Information on the study was explained to the participants, including the procedures, potential risks, and benefits of the study. Informed voluntary written and signed consent was obtained from all respondents prior to the study.

## 3. Result

A total of 489 patients were interviewed in this study with response rate of 99.4%. Out of the total 489 study subjects, females make a majority 300 (61.3%). One hundred sixty-six (33.9%) were aged between 25 and 34 years with mean age (±SD) 36.48 ± 11.6 years. Muslim were predominantly 361 (73.8%) in religion and majority of the respondents 302 (61.8%) were Oromo in ethnicity. Three hundred fourteen (64.2%) of the respondents were married. Regarding educational status 206 (42.1%) were unable to write and read (Table 1).

**Figure 1:**
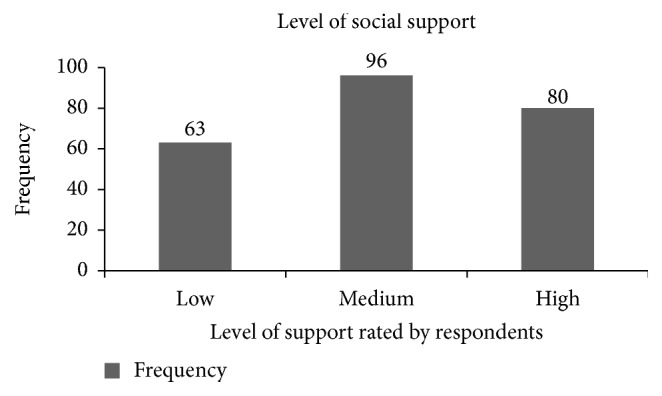
Frequency of level of social support of respondents on depression and associated factors among adult inpatients in Harari region hospitals, Eastern Ethiopia, 2017 (*n* = 239).

About 239 (48.9%) of the respondents got social support of which 96 (40.2%) rated that they received medium level of support (Figure 1). Majority 269 (55%) of the respondents stayed in the hospital for ≤1 week. One hundred thirteen (23.1%) had history of previous hospitalization and 118 (24.1%) were diagnosed with chronic morbidity, among which 28 (23.7%) had hypertension (Table 2). Among study subjects, 42 (8.6%) used alcohol, among which 20 (47.6%) used it for ≤5 years. Fifty-five (11.2%) were cigarette smokers, 24 (43.6%) of which used it for 6–10 years. Psychoactive drug users were 96 (19.6%) and majority 89 (92.7%) used Khat (Table 3).

**Figure 2:**
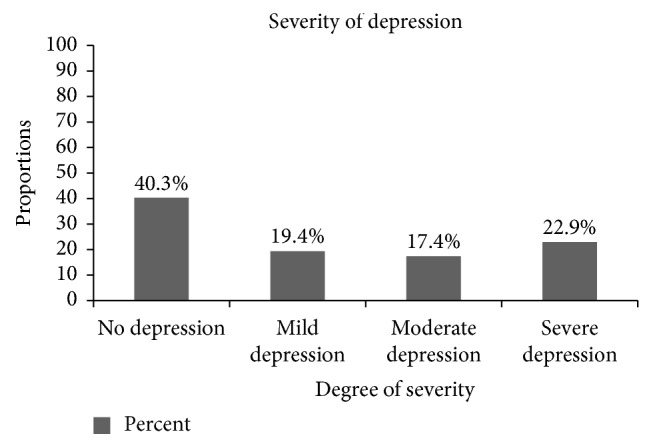
Severity of depression of respondents on depression and associated factors among adult inpatients in Harari region hospitals, Eastern Ethiopia, 2017 (*n* = 489).

The prevalence of depression among 489 adult patients admitted to governmental hospitals was 292 (59.7%; 95% CI = 55.7%, 64%). Out of the total patients with depression, 112 (22.9%) had severe depression (Figure 2).

Result of multivariable logistic regression indicated that having duration of 1-2 weeks in the hospital, being diagnosed with chronic morbidity, and being users of psychoactive drugs [AOR = 2.02, 95% CI: (1.28, 3.19); AOR = 4.06, 95% CI: (2.23, 7.40); and AOR = 2.24, 95% CI: (1.18, 4.24)] were more likely associated with development of depressive symptoms as compared with their counterparts, respectively, whereas patients who were admitted to surgical ward were less likely [AOR = 0.50, 95% CI: (0.31, 0.81)] to have depressive symptoms compared to those who were admitted to medical ward (Table 4).

## 4. Discussion

The prevalence of depression in this study was found to be 59.7% (95% CI = 55.7%, 64%); out of it 112 (22.9%) had severe depression and it is mainly affected by hospital stay, having chronic disease, and substance use.

The prevalence of depression in this study was higher than different studies done in Ethiopia with prevalence ranging from 38.3 to 58.6 [1, 15, 16]. Among the types of depression severe depression was higher compared to studies conducted in Gonder and Mekelle where it was 0.8% [17] and 17% [1], respectively. This difference might be due to the fact that in this study area psychoactive drugs use was high; in addition there might be methodological differences, measurement tool, and differences in socioeconomic environments between the studies.

This study revealed that being admitted to surgical ward was protective and is not consistent with that of Mekele study which might be due to hostile environment created between patient and care givers. But it needs further investigation.

Patients hospital stay was significantly associated with depression and this extends study in Gonder [16] in which patients who were stayed in hospital one to two weeks are two times more likely to develop depressive symptoms as compared with those who stayed for less than one week. Treatment adherence is known to be poor among patients with depression, leading patients to increasing length of stay. Also it aggravates chronic diseases and disability. This could increase unexplained physical symptoms and, consequently, increase hospital stay.

Depression was common among patients with chronic disease in study from Brazil and Jimma [18, 19], respectively, which is consistent with this study finding. This might be explained by the fact that depression is the most common consequences of physical health problems and people with chronic diseases can present with limitations like self-care deficit and activity of daily life.

Using psychoactive drug was associated with depression which was witnessed by the study from Brazil [20]. Our study was consistent with this finding which may be attributed to patients who are dependent on psychoactive drug and might discontinue the drug during hospital stay or the effect of disease might cause them to stop it.

To appreciate the findings of this survey, some potential limitations in the design and measurements need to be addressed. First this study did not use gold standard diagnostic criteria such as DSM IV for major depressive disorder; rather it relied on cutoff of PHQ-9 scores of ≥5 for point prevalence estimates for screened depression. This might have resulted in overestimates of point prevalence of screened cases. Secondly the cross-sectional nature of this study might limit causal association. Thirdly since the study was institutional based, it might not be generalized to the total population.

In conclusion, the prevalence of depression among admitted inpatients was high. Prolonged stay, diagnoses with chronic disease, and use of psychoactive drug were factors that are associated with development of depressive symptoms. Being admitted to surgical ward was related to low depression. Increasing the awareness of benefits of early diagnosis of patients to prevent major form of depression and cost saving of health system should be considered.

## Figures and Tables

**Table 1:** Sociodemographic characteristics of respondents on depression and associated factors among adult inpatients in Harari region hospitals, Eastern Ethiopia, 2017 (*n* = 489).

Variables	Frequency	Percentage
Age		
18–24	60	12.3
25–34	166	33.9
35–44	144	29.4
45–54	81	16.6
55–64	28	5.7
>65	10	2.0
Sex		
Male	189	38.7
Female	300	61.3
Religion		
Muslim	361	73.8
Orthodox	104	21.3
Other	24	4.9
Ethnicity		
Oromo	302	61.8
Harari	69	14.1
Amhara	78	16.0
Tigray	21	4.3
Others	19	3.9
Marital status		
Single	81	16.6
Married	314	64.2
Widowed	52	10.6
Divorced	42	8.6
Educational level		
Unable to write and read	206	42.1
Able to read and write	95	19.4
Attended primary school (1–8)	100	20.4
Attended secondary school (9–12)	45	9.2
College and above	43	8.8
Residence		
Urban	183	37.4
Rural	306	62.6
Occupation		
Farmer	99	20.2
House wife	210	42.9
Merchant	73	14.9
Government employee	50	10.2
Nongovernment employee	28	5.7
Others	29	5.9

**Table 2:** History of hospitalization and diagnoses related factors of respondents on depression and associated factors among adult inpatients in Harari region hospitals, Eastern Ethiopia, 2017.

Variables	Frequency	Percentage
Period of stay in hospital (*n* = 489)		
<1 week	269	55
1-2 weeks	180	36.8
≥3 weeks	40	8.2
Previous hospitalization (*n* = 489)		
Yes	113	23.1
No	376	76.9
Diagnosis with chronic condition (*n* = 489)		
Yes	118	24.1
No	371	75.9
Type of diagnosis (*n* = 118)		
Diabetes	19	16.1
Hypertension	28	23.7
COPD	20	16.9
Heart failure	16	13.6
TB	16	13.6
Others	19	16.1

**Table 3:** Substance uses of respondents on depression and associated factors among adult inpatients in Harari region hospitals, Eastern Ethiopia, 2017.

Types of substance	Frequency	Percent
Alcohol use (*n* = 489)		
Yes	42	8.6
No	447	91.4
Period of alcohol use (*n* = 42)		
≤5 years	20	47.6
6–10 years	12	28.6
≥11 years	10	23.8
Cigarette smoking (*n* = 489)		
Yes	55	11.2
No	434	88.8
Period of cigarette smoking (*n* = 55)		
≤5 years	10	18.2
6–10 years	24	43.6
≥11 years	21	38.2
Psychoactive drugs use (*n* = 489)		
Yes	96	19.6
No	393	80.4
Type of psychoactive drugs used (*n* = 96)		
Shisha	7	7.3
Khat	89	92.7

**Table 4:** Factors associated with depression among adult inpatients in Harari region hospitals, Eastern Ethiopia, 2017 (*n* = 489).

Independent variables	Frequency	Depression		COR (95% CI)	AOR (95% CI)
		Yes	No		
Sex					
Male	189	125	64	1.56 (1.07, 2.27)^*∗*^	1.12 (0.68, 1.84)
Female	300	167	133	1	1
Age					
18–24	60	29	31	1	1
25–34	166	81	73	1.19 (0.65, 2.16)	1.53 (0.70, 3.38)
35–44	144	90	50	1.84 (0.99, 3.40)	1.80 (0.77, 4.25)
45–54	81	60	21	3.05 (1.50, 6.21)^*∗*^	1.97 (0.77, 5.05)
55–64	28	25	12	2.23 (0.95, 5.23)	1.14 (0.37, 3.52)
>65	10	7	10	1.18 (0.44, 3.18)	0.38 (0.09, 1.66)
Marital status					
Single	81	45	36	1	1
Married	314	168	146	0.92 (0.56, 1.50)	0.73 (0.37, 1.47)
Widowed	52	44	8	4.40 (1.84, 10.52)^*∗*^	2.92 (0.98, 8.66)
Divorced	42	35	7	4.00 (1.59, 10.06)^*∗*^	2.81 (0.93, 8.44)
Educational status					
Unable to write & read	206	129	77	1	1
Able to read and write	95	56	39	0.86 (0.52, 1.41)	0.99 (0.56, 1.76)
Attended primary school (1–8)	100	48	52	0.55 (0.34, 0.89)^*∗*^	0.72 (0.40, 1.29)
Attended secondary school (9–12)	45	30	15	1.19 (0.60, 2.36)	1.62 (0.74, 3.55)
College and above	43	29	14	1.24 (0.62, 2.48)	1.66 (0.76, 3.66)
Ward admitted					
Medical	179	119	60	1	1
Surgical	186	105	81	0.65 (0.43, 0.10)^*∗*^	0.50 (0.31, 0.81)^*∗*^
Gynecology	124	68	56	0.61 (0.38, 0.98)^*∗*^	0.64 (0.36, 1.12)
Social support					
Yes	239	130	109	0.65 (0.45, 0.93)^*∗*^	0.70 (0.45, 1.08)
No	250	162	88	1	1
Previous hospital admission					
Yes	113	77	36	1.6 (1.03, 2.50)^*∗*^	1.16 (0.67, 2.00)
No	376	215	161	1	1
Period of stay in hospital					
<1 week	269	136	133	1	1
1-2 weeks	180	126	54	2.23 (1.53, 3.40)^*∗∗*^	2.02 (1.28, 3.19)^*∗*^
≥3 weeks	40	30	10	2.93 (1.38, 6.24)^*∗*^	2.08 (0.85, 5.05)
Dx with chronic condition					
Yes	118	100	18	5.18 (3.01, 8.90)^*∗∗*^	4.06 (2.23, 7.40)^*∗∗*^
No	371	192	179	1	1
Cigarette smoking					
Yes	55	40	15	1.93 (1.03, 3.59)^*∗*^	0.91 (0.40, 2.05)
No	434	252	182	1	1
Psychoactive drugs use					
Yes	96	74	22	2.70 (1.61, 4.52)^*∗∗*^	2.24 (1.18, 4.24)^*∗*^
No	393	218	175	1	1

^*∗*^ *p* value < 0.05; ^*∗∗*^*p* value < 0.001. CI: confidence interval, COR: crude odds ratio, AOR = adjusted odds ratio, and Dx = diagnosis.
